# Supplementary material for: Computational study on ratio-sensing in yeast galactose utilization pathway
Source: PLoS Comput Biol. 2020 Dec 4;16(12):e1007960. doi: 10.1371/journal.pcbi.1007960 (PMC7744065; doi:10.1371/journal.pcbi.1007960)
Supplement: S1 Text — (DOCX) [file pcbi.1007960.s001.docx]

# Supporting information for

# Computational study on ratio-sensing in yeast galactose utilization pathway

Jiayin Hong, Bo Hua, Michael Springer^*^, and Chao Tang^*^

* Corresponding author

E-mail: [michael_springer@hms.harvard.edu](mailto:michael_springer@hms.harvard.edu) (M.S.), tangc@pku.edu.cn (C.T.)

# S1 Text. Model derivations and descriptions

**Modeling competitive binding at transporter level**

Sugar transportation can be regarded as a two-step process. First, outside the cellular membrane, extracellular sugars bind to transporters with a forward binding rate ${kf}_{gal}$, forming ‘loaded’ transporters $\left[ gal\text{-}T \right]$ or $\left[ gluc\text{-}T \right]$, which could dissociate at rate ${kr}_{gal}$ (though comparatively rather small). Secondly, inside the cellular membrane, the loaded transporters unload sugars into cytoplasm at rate $k_{trans}$, releasing free transporters and intracellular sugars. Hence, the intracellular sugars are accumulated through transportation of extracellular sugars and balanced by carbon catabolism. We assumed that the total amount of membrane transporters was a constant, and that the transporters could be free, bound by galactose, or bound by glucose. Thus:

$$\begin{aligned} T_{total}=T_{free}+\left[ gal\text{-}T \right]+\left[ gluc\text{-}T \right]\#\left( S1 \right) \end{aligned}$$

Using the law of mass action, the levels of intracellular galactose, intracellular glucose, transporters bound by galactose, transporters bound by glucose, and free transporters are related as follows:

$$\begin{aligned} \frac{d{gal}_{in}}{dt}=k_{trans}\cdot\left[ gal\text{-}T \right]-\gamma\cdot{gal}_{in}\#\left( S2 \right) \end{aligned}$$

$$\begin{aligned} \frac{d{gluc}_{in}}{dt}=k_{trans}\cdot\left[ gluc\text{-}T \right]-\gamma\cdot{gluc}_{in}\#\left( S3 \right) \end{aligned}$$

$$\begin{aligned} \frac{d\left[ gal\text{-}T \right]}{dt}={kf}_{gal}\cdot{gal}_{ex}\cdot T_{free}-{kr}_{gal}\cdot\left[ gal\text{-}T \right]-k_{trans}\cdot\left[ gal\text{-}T \right]\#\left( S4 \right) \end{aligned}$$

$$\begin{aligned} \frac{d\left[ gluc\text{-}T \right]}{dt}={kf}_{gluc}\cdot{gluc}_{ex}\cdot T_{free}-{kr}_{gluc}\cdot\left[ gluc\text{-}T \right]-k_{trans}\cdot\left[ gluc\text{-}T \right]\#\left( S5 \right) \end{aligned}$$

$$\begin{aligned} \frac{dT_{free}}{dt}=k_{trans}\cdot\left[ gal\text{-}T \right]+k_{trans}\cdot\left[ gluc\text{-}T \right]+{kr}_{gal}\cdot\left[ gal\text{-}T \right]+{kr}_{gluc}\cdot\left[ gluc\text{-}T \right] \\ -{kf}_{gal}\cdot{gal}_{ex}\cdot T_{free}-{kf}_{gluc}\cdot{gluc}_{ex}\cdot T_{free}\#\left( S6 \right) \end{aligned}$$

When the above reactions reach steady state, the left sides of equations (S2) to (S6) are all equal to 0, and we can derive the levels of these species at steady state as shown in equations (S7) to (S11).

$$\begin{aligned} {gal}_{in}=\frac{k_{trans}}{\gamma}\cdot\left[ gal\text{-}T \right]\#\left（ S7 \right） \end{aligned}$$

$$\begin{aligned} {gluc}_{in}=\frac{k_{trans}}{\gamma}\cdot\left[ gluc\text{-}T \right]\#\left（ S8 \right） \end{aligned}$$

$$\begin{aligned} \left[ gal\text{-}T \right]=\frac{{kf}_{gal}\cdot{gal}_{ex}}{{kr}_{gal}+k_{trans}}\cdot T_{free}\#\left( S9 \right) \end{aligned}$$

$$\begin{aligned} \left[ gluc\text{-}T \right]=\frac{{kf}_{gluc}\cdot{gluc}_{ex}}{{kr}_{gluc}+k_{trans}}\cdot T_{free}\#\left( S10 \right) \end{aligned}$$

$$\begin{aligned} T_{free}=\frac{{kr}_{gal}\cdot\left[ gal\text{-}T \right]+{kr}_{gluc}\cdot\left[ gluc\text{-}T \right]+k_{trans}\cdot\left[ gal\text{-}T \right]+k_{trans}\cdot\left[ gluc\text{-}T \right]}{{kf}_{gal}\cdot{gal}_{ex}+{kf}_{gluc}\cdot{gluc}_{ex}}\#\left( S11 \right) \end{aligned}$$

Let $K_{gal}=\frac{{kr}_{gal}+k_{trans}}{{kf}_{gal}}，K_{gluc}=\frac{{kr}_{gluc}+k_{trans}}{{kf}_{gluc}}$, and then substitute equations (S9) to (S11) into equation (S1), yielding

$$\begin{aligned} T_{free}=\frac{T_{total}}{1+\frac{{gal}_{ex}}{K_{gal}}+\frac{{gluc}_{ex}}{K_{gluc}}}\#\left( S12 \right) \end{aligned}$$

Substituting equations (S9) and (S12) into equation (S7), we can obtain

$$\begin{aligned} {gal}_{in}&=\frac{k_{trans}}{\gamma}\cdot\left[ gal\text{-}T \right] \\ &=\frac{k_{trans}}{\gamma}\cdot\frac{{kf}_{gal}\cdot{gal}_{ex}}{{kr}_{gal}+k_{trans}}\cdot T_{free} \\ &=\frac{k_{trans}}{\gamma}\cdot\frac{{gal}_{ex}}{K_{gal}}\cdot\frac{T_{total}}{1+\frac{{gal}_{ex}}{K_{gal}}+\frac{{gluc}_{ex}}{K_{gluc}}} \\ &=\frac{k_{trans}}{\gamma}\cdot\frac{T_{total}}{\frac{K_{gal}}{{gal}_{ex}}+1+\frac{{gluc}_{ex}}{{gal}_{ex}}\cdot\frac{K_{gal}}{K_{gluc}}}\#(S13) \end{aligned}$$

Equation (S13) is equivalent to equation (1) in the main text, and by similar derivation we obtained equation (7) in the main text.

As shown in the main text, the intracellular galactose level at steady state is

$$\begin{aligned} {gal}_{in}=\frac{k_{trans}}{\gamma}\cdot T_{total}\cdot\frac{1}{1+\frac{K_{gal}}{{gal}_{ex}}\cdot\left( 1+\frac{{gluc}_{ex}}{K_{gluc}} \right)}\#\left( 1 \right) \end{aligned}$$

where $K_{gal}=\frac{{kr}_{gal}+k_{trans}}{{kf}_{gal}}$ and $K_{gluc}=\frac{{kr}_{gluc}+k_{trans}}{{kf}_{gluc}}$ are the binding coefficients for galactose and glucose, respectively. $k_{trans}$ is the maximal transportation rate through the given transporter, $\gamma$ is the turn-over rate of the sugar within a cell, $T_{total}$ is the total number of transporters expressed on the membrane. When these three parameters are fixed in a cell, the external galactose and glucose concentrations jointly determined intracellular galactose level. In a minimal and general transporter regulatory model, we circumvented transcriptional regulatory circuit consisting of Gal3p, Gal80p, and Gal4p. Instead, we proposed a simple yet reasonable assumption for induction of the GAL pathway, which linearly depends on intracellular galactose level. Thus, the contour lines of identical intracellular galactose levels correspond to different induction levels of the pathway in Fig. 2.

To derive the expression of the contour lines and solve for the slope and the intercept on *gal* titration axis, we set the fraction in equation (1) equal to a constant (*const*):

$$\begin{aligned} \frac{1}{1+\frac{K_{gal}}{{gal}_{ex}}\cdot\left( 1+\frac{{gluc}_{ex}}{K_{gluc}} \right)}=const\#\left( S14 \right) \end{aligned}$$

For instance, when the *const* equals to 0.5, it means that the intracellular galactose level reaches half maximum, corresponding to a half-maximum decision front of the GAL pathway induction.

When ${gluc}_{ex}\gg K_{gluc}$, equation (S14) in log-log scale is approximately:

$$\begin{aligned} \log{gluc}_{ex}=\log{gal}_{ex} +\log\frac{K_{gluc}}{K_{gal}}+const'\#\left（ S15 \right） \end{aligned}$$

where $const^{'}=log(\frac{1}{const}-1)$. A generalized form of equation (S15) incorporating cooperativity was shown in equation (2) in the main text.

**Modeling competitive inhibition at transcriptional level**

We assumed that GAL1 gene copies within a cell are constant, and that the cis-regulatory element (CRE) of GAL1 could be bound by the activator, bound by the repressor, or free. So, we have,

$$\begin{aligned} {GAL1}_{\mathrm{total}}={GAL1}_{\mathrm{free}}+{GAL1}_{\mathrm{active}}+{GAL1}_{\mathrm{repressed}}\#\left( S16 \right) \end{aligned}$$

The association and dissociation between the sugars and the sensors are as follows

$$\begin{aligned} \frac{d {Activator}^{*}}{dt}={kf}_{G}\cdot{gal}_{in}\cdot\left( {Activator}_{total}-{Activator}^{*} \right)-{kr}_{G}\cdot{Activator}^{*}\#\left( S17 \right) \end{aligned}$$

$$\begin{aligned} \frac{d {Repressor}^{*}}{dt}={kf}_{M}\cdot{gluc}_{in}\cdot\left( {Repressor}_{total}-{Repressor}^{*} \right)-{kr}_{M}\cdot{Repressor}^{*}\#\left( S18 \right) \end{aligned}$$

The mass actions between the activator, the repressor, and the CRE are as follows

$$\begin{aligned} \frac{d {GAL1}_{\mathrm{active}}}{\mathrm{dt}}=\mathrm{kf}_{A}\cdot{GAL1}_{\mathrm{free}}\cdot\mathrm{Activator}^{*}-\mathrm{kr}_{A}\cdot{GAL1}_{\mathrm{active}}\#\left( S19 \right) \end{aligned}$$

$$\begin{aligned} \frac{d {GAL1}_{\mathrm{repressed}}}{\mathrm{dt}}=\mathrm{kf}_{R}\cdot{GAL1}_{\mathrm{free}}\cdot\mathrm{Repressor}^{*}-\mathrm{kr}_{R}\cdot{GAL1}_{\mathrm{repressed}}\#\left( S20 \right) \end{aligned}$$

When the association and dissociation between regulatory factors and CRE reach equilibrium, we have,

$$\begin{aligned} {GAL1}_{\mathrm{active}}=\frac{\mathrm{Activator}^{*}}{K_{A}}\cdot{GAL1}_{\mathrm{free}}\#\left( S21 \right) \end{aligned}$$

$$\begin{aligned} {GAL1}_{\mathrm{repressed}}=\frac{\mathrm{Repressor}^{*}}{K_{R}}\cdot{GAL1}_{\mathrm{free}}\#\left( S22 \right) \end{aligned}$$

where $K_{A}=\frac{{kr}_{A}}{{kf}_{A}}$，$K_{R}=\frac{{kr}_{R}}{{kf}_{R}}$.

If all the GAL1 gene copies are bound by the activator and thus transcriptionally activated, then the pathway is in its maximum induction. We approximated the induction levels by calculating the actively transcribed GAL1 gene fraction.

$$\begin{aligned} \frac{{GAL1}_{active}}{{GAL1}_{total}}&=\frac{{GAL1}_{active}}{{GAL1}_{active}+{GAL1}_{repressed}+{GAL1}_{free}} \\ &=\frac{\frac{\mathrm{Activator}^{*}}{K_{A}}\cdot{GAL1}_{\mathrm{free}}}{\frac{\mathrm{Activator}^{*}}{K_{A}}\cdot{GAL1}_{\mathrm{free}}+\frac{\mathrm{Repressor}^{*}}{K_{R}}\cdot{GAL1}_{free}+{GAL1}_{free}} \\ &=\frac{1}{1+\frac{\mathrm{Repressor}^{*}}{\mathrm{Activator}^{*}}\cdot\frac{K_{A}}{K_{R}}+\frac{K_{A}}{\mathrm{Activator}^{*}}} \\ &=\frac{1}{1+\frac{K_{A}}{\mathrm{Activator}^{*}}\cdot\left( 1+\frac{\mathrm{Repressor}^{*}}{K_{R}} \right)}\#(S23) \end{aligned}$$

Substitute equations (S3) and (S4) to equation (S23), we have

$\begin{aligned} \frac{{GAL1}_{active}}{{GAL1}_{total}}=\frac{1}{1+\frac{K_{A}}{{Activator}_{total}}\cdot\left( 1+\frac{K_{G}}{{gal}_{\mathrm{in}}} \right)\cdot\left( 1+\frac{{Repressor}_{total}}{K_{R}\cdot\left( 1+\frac{K_{M}}{{gluc}_{\mathrm{in}}} \right)} \right)}\#\left( S24 \right) \end{aligned}$which is equivalent to equation (5) in the main text, where $\emptyset_{A}=\frac{{Activator}_{total}}{K_{A}}$，and $\emptyset_{R}=\frac{{Repressor}_{total}}{K_{R}}$.

To obtain the formula for decision front in the transcriptional level model, i.e. equation (6) in the main text, we followed similar procedures as that in the transporter model. We set equation (5) equal to a constant (*const*):

$$\begin{aligned} \frac{1}{1+\frac{1}{\emptyset_{A}}\cdot\left( \frac{K_{G}}{{gal}_{in}}+1 \right)\cdot\left( 1+\frac{\emptyset_{R}}{\frac{K_{M}}{{gluc}_{in}}+1} \right)}=const\#\left( S25 \right) \end{aligned}$$

Again, when the *const* equals to 0.5, it means that half of all the GAL1 gene copies are actively transcribed, corresponding to a half-maximum decision front of the GAL pathway induction.

When ${gal}_{in}\ll K_{G}，\frac{K_{M}}{\emptyset_{R}}\ll{gluc}_{in}\ll K_{M}$, equation (S25) is approximately:

$$\begin{aligned} \frac{1}{1+\frac{1}{\emptyset_{A}}\cdot\frac{K_{G}}{{gal}_{in}}\cdot\frac{\emptyset_{R}}{K_{M}}\cdot{gluc}_{in}}=const\#\left( S26 \right) \end{aligned}$$

which in log-log scale is:

$$\begin{aligned} \log{gluc}_{in}=\log{gal}_{in} +\log\frac{K_{M}}{K_{G}}+\log\frac{\emptyset_{A}}{\emptyset_{R}}+const'\#\left（ 6 \right） \end{aligned}$$

where $const^{'}=log(\frac{1}{const}-1)$.

**Introducing network motifs to transcriptional level model**

The generalized expression for the actively transcribed fraction of given gene copies is,

$$\begin{aligned} \mathrm{frac}_{\mathrm{active}}=\frac{1}{1+\frac{K_{A}}{A_{\mathrm{total}}}\cdot\left( \frac{K_{G}}{S_{A}}+1 \right)\cdot\left( 1+\frac{R_{\mathrm{total}}}{K_{R}}\cdot\frac{S_{R}}{K_{M}+S_{R}} \right)}\#\left( S27 \right) \end{aligned}$$

where $S_{A}$ and $S_{R}$ represent the concentration of activating input signal and of repressing input signal, respectively. In the case of GAL pathway induction, $S_{A}$ corresponds to galactose and $S_{R}$ corresponds to glucose.

1. Negative auto-regulation at the activator node

We first considered introducing negative auto-regulation to the activator node, which means that making more activators would lead to inhibition of activator expression. Consider the rate of synthesis of the activator is repressed by its functional form $A^{*}$, balanced by degradation/dilution of the protein at rate $\alpha$, so that:

$$\begin{aligned} \frac{dA_{\mathrm{total}}}{\mathrm{dt}}=\beta\cdot\frac{K_{x}}{K_{x}+A^{*}}-\alpha\cdot A_{\mathrm{total}}\#\left( S28 \right) \end{aligned}$$

where $A^{*}$ is the functional form of the activator upon binding by $S_{A}$, and $K_{x}$ is the dissociation constant of the activator from its own promoter. Substituting equation (3) into equation (S28) and assuming strong binding of the activator to its own promoter $K_{x}\ll A^{*}$, at steady state, we found

$$\begin{aligned} A_{\mathrm{total}}\approx A_{0}\cdot\frac{K_{x}}{\frac{S_{A}}{K_{G}+S_{A}}\cdot A_{total}} \\ \Longrightarrow A_{\mathrm{total}}=\sqrt{A_{0}\cdot K_{x}\cdot\left( \frac{K_{G}}{S_{A}}+1 \right)}\#\left( S29 \right) \end{aligned}$$

where $A_{0}=\frac{\beta}{\alpha}$, the expression level of the activator without introducing negative auto-regulation. Within the ratio-sensing regime, substituting equation (S29) to equation (S27) yields

$$\begin{aligned} \mathrm{frac}_{\mathrm{active}}=\frac{1}{1+\frac{K_{A}}{K_{R}}\cdot\frac{\sqrt{K_{G}}}{K_{M}}\cdot\frac{R_{\mathrm{total}}}{\sqrt{{A_{0}\cdot K}_{x}}}\cdot\frac{S_{R}}{\sqrt{S_{A}}}}\#\left( S30 \right) \end{aligned}$$

Meanwhile, we could also solve the formula for the decision front as follows:

$$\begin{aligned} \log S_{R}=\frac{1}{2}\log S_{A} +\log\left( \frac{K_{M}}{\sqrt{K_{G}}}\cdot\frac{K_{R}}{K_{A}}\cdot\frac{\sqrt{A_{0}\cdot K_{x}}}{R_{\mathrm{total}}} \right)+const\#\left( S31 \right) \end{aligned}$$

Equation (S31) indicates that incorporating negative auto-regulation at the activator node would halve the slope of the decision front in log-log scale, in other words, the network would become more sensitive to the inhibiting glucose signal. The intercept on the galactose titration axis also becomes dependent on the square root of $K_{G}$ and $A_{0}$, which means that the decision to induce the network is more robust to variation of the binding affinity between galactose and the activator, as well as more robust to the fluctuation of the activator level.

1. Positive auto-regulation at the activator node

Next, we considered the introduction of positive auto-regulation to the activator node. This gives

$$\begin{aligned} \frac{dA_{\mathrm{total}}}{\mathrm{dt}}=\beta\cdot\frac{A^{*}}{K_{x}+A^{*}}-\alpha\cdot A_{\mathrm{total}}\#\left( S32 \right) \end{aligned}$$

At steady state,

$$\begin{aligned} A_{\mathrm{total}}=A_{0}\cdot\frac{1}{\frac{K_{x}}{A^{*}}+1}=A_{0}\cdot\frac{1}{\frac{K_{x}}{\frac{S_{A}}{K_{G}+S_{A}}\cdot A_{total}}+1} \\ \Longrightarrow A_{\mathrm{total}}=A_{0}-K_{x}\cdot\left( \frac{K_{G}}{S_{A}}+1 \right)\#\left( S33 \right) \end{aligned}$$

Within the ratio-sensing regime, substituting equation (S33) to equation (S27) yields

$$\begin{aligned} \mathrm{frac}_{\mathrm{active}}=\frac{1}{1+\frac{K_{A}}{K_{R}}\cdot\frac{K_{G}}{K_{M}}\cdot\frac{R_{\mathrm{total}}}{{A_{0}-K}_{x}\cdot K_{G}\cdot\frac{1}{S_{A}}}\cdot\frac{S_{R}}{S_{A}}}\#\left( S34 \right) \end{aligned}$$

And the formula for the decision front is

$$\begin{aligned} \log S_{R}=\log\left( S_{A}-\frac{K_{x}\cdot K_{G}}{A_{0}} \right) +\log\left( \frac{K_{M}}{K_{G}}\cdot\frac{K_{R}}{K_{A}}\cdot\frac{A_{0}}{R_{\mathrm{total}}} \right)+const\#\left（ S35 \right） \end{aligned}$$

Equation (S35) suggests that incorporating positive auto-regulation to the activator changes neither the slope of the decision front, nor the sensitivity to input signals.

1. Negative auto-regulation at the repressor node

When negative auto-regulation of the repressor is introduced,

$$\begin{aligned} \frac{dR_{\mathrm{total}}}{\mathrm{dt}}=\beta\cdot\frac{K_{y}}{K_{y}+R^{*}}-\alpha\cdot R_{\mathrm{total}}\#\left( S36 \right) \end{aligned}$$

where $R^{*}$ represents the functional form of the repressor upon binding by glucose and $K_{y}$ is the dissociation constant of the repressor from its own promoter. Again, we solved for total repressor when the system reaches steady state:

$$\begin{aligned} R_{\mathrm{total}}\approx R_{0}\cdot\frac{K_{y}}{\frac{S_{R}}{K_{M}+S_{R}}\cdot R_{total}} \\ \Longrightarrow R_{\mathrm{total}}=\sqrt{R_{0}\cdot K_{y}\cdot\left( \frac{K_{M}}{S_{R}}+1 \right)}\#\left( S37 \right) \end{aligned}$$

where $R_{0}=\frac{\beta}{\alpha}$, the expression level of the repressor without the negative auto-regulation motif. Within the ratio-sensing regime, substituting equation (S37) to equation (S27) yields

$$\begin{aligned} \mathrm{frac}_{\mathrm{active}}=\frac{1}{1+\frac{K_{A}}{K_{R}}\cdot\frac{K_{G}}{\sqrt{K_{M}}}\cdot\frac{\sqrt{{R_{0}\cdot K}_{y}}}{A_{\mathrm{total}}}\cdot\frac{\sqrt{S_{R}}}{S_{A}}}\#\left( S38 \right) \end{aligned}$$

The formula for the decision front is

$$\begin{aligned} \log S_{R}=2\log S_{A} +\log\left( \frac{K_{M}}{K_{G}^{2}}\cdot\frac{K_{R}^{2}}{K_{A}^{2}}\cdot\frac{A_{\mathrm{total}}^{2}}{R_{0}\cdot K_{y}} \right)+const\#\left（ S39 \right） \end{aligned}$$

Equation (S39) implies that incorporating negative auto-regulation at the repressor node doubles the slope of the decision front, which means that the network becomes more sensitive to the activating galactose signal. Meanwhile, the intercept on the galactose titration axis becomes dependent on the square of $K_{G}$, $K_{R}$, $K_{A}$ and $A_{total}$, so that the decision to induce the network is more susceptible to variations in the binding affinity between galactose and the activator, or the binding affinity between the regulator and the cis-regulatory element of GAL1, as well as more sensitive to fluctuations in the activator level.

1. Positive auto-regulation at the repressor node

Similar to the activator node, introducing positive auto-regulation to the repressor node does not change the slope of the decision front. The actively transcribed gene fraction and the formula for the decision front are given by:

$$\begin{aligned} \mathrm{frac}_{\mathrm{active}}=\frac{1}{1+\frac{K_{A}}{K_{R}}\cdot\frac{K_{G}}{K_{M}}\cdot\frac{{R_{0}-K}_{y}\cdot K_{M}\cdot\frac{1}{S_{R}}}{A_{\mathrm{total}}}\cdot\frac{S_{R}}{S_{A}}}\#\left( S40 \right) \end{aligned}$$

$$\begin{aligned} \log\left( S_{R}-\frac{K_{y}\cdot K_{M}}{R_{0}} \right)=\log S_{A} +\log\left( \frac{K_{M}}{K_{G}}\cdot\frac{K_{R}}{K_{A}}\cdot\frac{A_{\mathrm{total}}}{R_{0}} \right)+const\#\left（ S41 \right） \end{aligned}$$

1. Type I incoherent feedforward loop (the activator promotes the repressor)

We next sought to understand how feedforward loops change the integration of the different signals. First, consider the case where the activator promotes the expression of the repressor, forming a type I incoherent feedforward loop (IFFL type I). We have

$$\begin{aligned} \frac{dR_{\mathrm{total}}}{\mathrm{dt}}=\beta\cdot\frac{A^{*}}{K_{y}+A^{*}}-\alpha\cdot R_{\mathrm{total}}\#\left( S42 \right) \end{aligned}$$

where $K_{y}$ is the dissociation constant of the activator from the promoter of the repressor. When cells reach steady state:

$$\begin{aligned} R_{\mathrm{total}}&=R_{0}\cdot\frac{1}{\frac{K_{y}}{A^{*}}+1}=R_{0}\cdot\frac{1}{\frac{K_{y}}{\frac{S_{A}}{K_{G}+S_{A}}\cdot A_{total}}+1} \\ &=\frac{R_{0}}{1+\frac{K_{y}}{A_{\mathrm{total}}}\cdot\left( \frac{K_{G}}{S_{A}}+1 \right)}\#\left( S43 \right) \end{aligned}$$

Within the ratio-sensing regime, substituting equation (S43) to equation (S27) yields

$$\begin{aligned} \mathrm{frac}_{\mathrm{active}}=\frac{1}{1+\frac{K_{A}}{K_{R}}\cdot\frac{K_{G}}{K_{M}}\cdot\frac{R_{0}\cdot S_{R}}{A_{\mathrm{total}}\cdot S_{A}+K_{y}\cdot K_{G}}}\#\left( S44 \right) \end{aligned}$$

Solving the formula for the decision front:

$$\begin{aligned} \log S_{R}=\log\left( S_{A}+\frac{K_{y}\cdot K_{G}}{A_{\mathrm{total}}} \right) +\log\left( \frac{K_{M}}{K_{G}}\cdot\frac{K_{R}}{K_{A}}\cdot\frac{A_{\mathrm{total}}}{R_{0}} \right)+const\#\left（ S45 \right） \end{aligned}$$

We found that introducing IFFL type I did not change the network sensitivity to input signals.

1. Type I coherent feedforward loop (the activator inhibits the repressor)

We next considered the case where the activator inhibited the expression of the repressor, forming type I coherent feedforward loop (CFFL type I). We have

$$\begin{aligned} \frac{dR_{\mathrm{total}}}{\mathrm{dt}}=\beta\cdot\frac{K_{y}}{K_{y}+A^{*}}-\alpha\cdot R_{\mathrm{total}}\#\left( S46 \right) \end{aligned}$$

where $K_{y}$ is the dissociation constant of the activator from the promoter of the repressor. And the total amount of the repressor at steady state:

$$\begin{aligned} R_{total}{=R}_{0}\cdot\frac{1}{\frac{A^{*}}{K_{y}}+1}=\frac{R_{0}}{1+\frac{A_{total}}{K_{y}}\cdot\frac{S_{A}}{K_{G}+S_{A}}}\#\left( S47 \right) \end{aligned}$$

Within the ratio-sensing regime, substituting equation (S47) to equation (S27) yields

$$\begin{aligned} {frac}_{active}=\frac{1}{1+\frac{K_{A}}{K_{R}}\cdot\frac{K_{G}^{2}}{K_{M}}\cdot\frac{R_{0}\cdot K_{y}}{A_{total}^{2}}\cdot\frac{S_{R}}{S_{A}^{2}}}\#\left( S48 \right) \end{aligned}$$

And the formula for the decision front:

$$\begin{aligned} \log S_{R}=2\log S_{A} +log\left( \frac{K_{M}}{K_{G}^{2}}\cdot\frac{K_{R}}{K_{A}}\cdot\frac{A_{total}^{2}}{R_{0}\cdot K_{y}} \right)+const\#\left（ S49 \right） \end{aligned}$$

Equation (S49) indicates that incorporating CFFL type I doubles the slope of the decision front in log-log scale, in other words, the network becomes more sensitive to the activating galactose signal. Meanwhile, the intercept on the galactose titration axis becomes dependent on the square of $K_{G}$ and $A_{\mathrm{total}}$, which means the decision to induce the network is more susceptible to variations in the binding affinity between galactose and the activator, as well as more sensitive to fluctuations in the activator level.

1. Type II incoherent feedforward loop (the repressor promotes the activator)

We then introduced an activating edge from the repressor to the activator, forming a type II incoherent feedforward loop (IFFL type II). The dynamical differential equation and steady state level of the activator are:

$$\begin{aligned} \frac{dA_{total}}{dt}=\beta\cdot\frac{R^{*}}{K_{x}+R^{*}}-\alpha\cdot A_{total}\#\left( S50 \right) \end{aligned}$$

$$\begin{aligned} A_{total}&=A_{0}\cdot\frac{1}{\frac{K_{x}}{R^{*}}+1}=A_{0}\cdot\frac{1}{\frac{K_{x}}{\frac{S_{R}}{K_{M}+S_{R}}\cdot R_{total}}+1} \\ &=\frac{A_{0}}{1+\frac{K_{x}}{R_{total}}\cdot\left( \frac{K_{M}}{S_{R}}+1 \right)}\#\left( S51 \right) \end{aligned}$$

Note that $K_{x}$ is the dissociation constant of the repressor from the promoter of the activator. Within the ratio-sensing regime, substituting equation (S51) to equation (S27) yields

$$\begin{aligned} {frac}_{active}=\frac{1}{1+\frac{K_{A}}{K_{R}}\cdot\frac{K_{G}}{K_{M}}\cdot\frac{R_{total}\cdot S_{R}+K_{x}\cdot K_{M}}{A_{0}\cdot S_{A}}}\#\left( S52 \right) \end{aligned}$$

And the formula of the decision front:

$$\begin{aligned} \log\left( S_{R}+\frac{K_{x}\cdot K_{M}}{R_{total}} \right)=\log S_{A} +log\left( \frac{K_{M}}{K_{G}}\cdot\frac{K_{R}}{K_{A}}\cdot\frac{A_{0}}{R_{total}} \right)+const\#\left( S53 \right) \end{aligned}$$

Equation (S53) indicates that incorporating IFFL type II does not change the network sensitivity to input signals.

1. Type II coherent feedforward loop (the repressor inhibits the activator)

Finally, we introduced an inhibitory edge from the repressor to the activator, forming a type II coherent feedforward loop (CFFL type II). The dynamical differential equation and steady state level of the activator are:

$$\begin{aligned} \frac{dA_{total}}{dt}=\beta\cdot\frac{K_{x}}{K_{x}+R^{*}}-\alpha\cdot A_{total}\#\left( S54 \right) \end{aligned}$$

$$\begin{aligned} A_{total}{=A}_{0}\cdot\frac{1}{\frac{R^{*}}{K_{x}}+1}=\frac{A_{0}}{1+\frac{R_{total}}{K_{x}}\cdot\frac{S_{R}}{K_{M}+S_{R}}}\#\left( S55 \right) \end{aligned}$$

Within the ratio-sensing regime, substituting equation (S55) to equation (S27) yields

$$\begin{aligned} {frac}_{active}=\frac{1}{1+\frac{K_{A}}{K_{R}}\cdot\frac{K_{G}}{K_{M}^{2}}\cdot\frac{R_{total}^{2}}{K_{x}\cdot A_{0}}\cdot\frac{S_{R}^{2}}{S_{A}}}\#\left( S56 \right) \end{aligned}$$

And the formula of the decision front:

$$\begin{aligned} \log S_{R}=\frac{1}{2}\log S_{A} +log\left( \frac{K_{M}}{\sqrt{K_{G}}}\cdot\sqrt{\frac{K_{R}}{K_{A}}}\cdot\frac{\sqrt{A_{0}\cdot K_{x}}}{R_{total}} \right)+const\#\left（ S57 \right） \end{aligned}$$

Equation (S57) implies that incorporating CFFL type II halves the slope of the decision front in log-log scale, in other words, the network is more sensitive to the inhibiting glucose signal. The intercept on the galactose titration axis is dependent on the square roots of $K_{G}$, $K_{R}$, $K_{A}$ and $A_{0}$, which means that the decision to induce the network is more robust to variations in the binding affinity between galactose and the activator, and between the regulators and the cis-regulatory element, as well as more robust to the fluctuation of the activator level.
