# Supplementary material for: Computational study on ratio-sensing in yeast galactose utilization pathway
Source: PLoS Comput Biol. 2020 Dec 4;16(12):e1007960. doi: 10.1371/journal.pcbi.1007960 (PMC7744065; doi:10.1371/journal.pcbi.1007960)
Supplement: S1 Table — (DOCX) [file pcbi.1007960.s002.docx]

# Supporting information for

# Computational study on ratio-sensing in yeast galactose utilization pathway

Jiayin Hong, Bo Hua, Michael Springer^*^, and Chao Tang^*^

* Corresponding author

E-mail: [michael_springer@hms.harvard.edu](mailto:michael_springer@hms.harvard.edu) (M.S.), tangc@pku.edu.cn (C.T.)

# S1 Table

| Parameter | Description | Units |
| --- | --- | --- |
| $\boldsymbol{K}_{\boldsymbol{gal}}$ | binding coefficient of galactose to the shared transporter | nM |
| $\boldsymbol{K}_{\boldsymbol{gluc}}$ | binding coefficient of glucose to the shared transporter | nM |
| $\boldsymbol{n}_{\boldsymbol{gal}}$ | cooperativity coefficient of galactose binding to the shared transporter | Dimensionless |
| $\boldsymbol{n}_{\boldsymbol{gluc}}$ | cooperativity coefficient of glucose binding to the shared transporter | Dimensionless |
| $\boldsymbol{K}_{\boldsymbol{G}}$ | binding coefficient of galactose to the activator | nM |
| $\boldsymbol{K}_{\boldsymbol{M}}$ | binding coefficient of glucose to the repressor | nM |
| $\boldsymbol{\phi}_{\boldsymbol{R}}$ | total repressor divided by dissociation constant of the repressor | Dimensionless |
| $\boldsymbol{\phi}_{\boldsymbol{A}}$ | total activator divided by dissociation constant of the activator | Dimensionless |

**S1 Table: Parameter descriptions and units.**
