# Supplementary material for: Computational study on ratio-sensing in yeast galactose utilization pathway
Source: PLoS Comput Biol. 2020 Dec 4;16(12):e1007960. doi: 10.1371/journal.pcbi.1007960 (PMC7744065; doi:10.1371/journal.pcbi.1007960)
Supplement: S4 Table — (DOCX) [file pcbi.1007960.s005.docx]

# Supporting information for

# Computational study on ratio-sensing in yeast galactose utilization pathway

Jiayin Hong, Bo Hua, Michael Springer^*^, and Chao Tang^*^

* Corresponding author

E-mail: [michael_springer@hms.harvard.edu](mailto:michael_springer@hms.harvard.edu) (M.S.), tangc@pku.edu.cn (C.T.)

# S4 Table

**S4 Table: Parameter values used in simulations for Fig 4c.** Each box corresponds to one of the subplots in Fig 4c.
