# Supplementary material for: Computational study on ratio-sensing in yeast galactose utilization pathway
Source: PLoS Comput Biol. 2020 Dec 4;16(12):e1007960. doi: 10.1371/journal.pcbi.1007960 (PMC7744065; doi:10.1371/journal.pcbi.1007960)
Supplement: S5 Table — (DOCX) [file pcbi.1007960.s006.docx]

# Supporting information for

# Computational study on ratio-sensing in yeast galactose utilization pathway

Jiayin Hong, Bo Hua, Michael Springer^*^, and Chao Tang^*^

* Corresponding author

E-mail: [michael_springer@hms.harvard.edu](mailto:michael_springer@hms.harvard.edu) (M.S.), tangc@pku.edu.cn (C.T.)

# S5 Table

| Parameter | Description | Value | Units |
| --- | --- | --- | --- |
| $\boldsymbol{\beta}_{\boldsymbol{1}}$ | production rate for activator (or repressor) | 1000 | nM/min |
| $\boldsymbol{\beta}_{\boldsymbol{2}}$ | production rate for repressor (or activator) | 10 | nM/min |
| $\boldsymbol{\alpha}$ | degradation rate for activator and repressor | 0.001 | 1/min |
| $\boldsymbol{K}_{\boldsymbol{A}}$ | dissociation constant for activator binding to upstream activation sequence | 1 | nM |
| $\boldsymbol{K}_{\boldsymbol{R}}$ | dissociation constant for repressor binding to upstream repression sequence | 1 | nM |
| $\boldsymbol{K}_{\boldsymbol{G}}$ | binding coefficient of galactose to the activator | ${10}^{9}$ | nM |
| $\boldsymbol{K}_{\boldsymbol{M}}$ | binding coefficient of glucose to the repressor | ${10}^{9}$ | nM |
| $\boldsymbol{K}_{\boldsymbol{x}}$ | auto-regulation Michaelis constant for activator | 1 | nM |
| $\boldsymbol{K}_{\boldsymbol{y}}$ | auto-regulation Michaelis constant for repressor | 1 | nM |

**S5 Table: Parameter values used in simulations for Fig 5.**
